# Dataset on the assessment of pervious concrete containing palm oil kernel shell and seashell in heavy metal removal from stormwater

**DOI:** 10.1016/j.dib.2023.109570

**Published:** 2023-09-14

**Authors:** Elnaz Khankhaje, Taehoon Kim, Hyounseung Jang, Chang-Soo Kim, Jimin Kim, Mahdi Rafieizonooz

**Affiliations:** a Architectural Engineering Program, School of Architecture, Seoul National University of Science and Technology, 232 Gongneung-ro, Gongneung-dong, Nowon-gu, Seoul, 01811, South Korea; b School of Civil and Environmental Engineering, Yonsei University, Yonsei-ro 50, Seodaemun-gu, Seoul 03722, South Korea

**Keywords:** Water quality, Pervious concrete, Heavy metal removal, Palm oil kernel shell, Seashell, Permeable pavement

## Abstract

The dataset currently available comprises data on the removal rates of heavy metals (Ba, Se, Cr, Fe, Cd, As, and Co) through the incorporation of seashells and palm oil kernel shells into pervious concrete for stormwater treatment. Stormwater runoff was collected from commercial areas in Taman University, Skudai, Johor, Malaysia. The stormwater samples underwent filtration and were preserved in high-density polyethylene (HDPE) bottles at a temperature of 4 °C for use as incoming water. The outgoing water, referred to as effluent, was obtained from tests performed on pervious concrete samples after a curing period of 28 days. The pervious concrete mixes were created with a water-to-binder ratio (w/b ratio) of 32% and a sand ratio of 10%. Three different levels of palm oil kernel shell and seashell content were used as coarse aggregate replacements: 0%, 25%, and 50%. Two single-size group were considered for both palm oil kernel shell and seashell: (6.3-9.5 mm) and (4.75-6.3 mm). Heavy metal analyses were conducted on the influent and effluent using a PerkinElmer ELAN 6100 Series Inductively Coupled Plasma– Mass Spectrometer (ICP-MS). The available datasets consist of both raw and analyzed data.

Specifications Table

**Table:** 

Subject	Environmental science

Specific subject area	Heavy metal removal by pervious concrete
Type of data	Table Figure
How the data were acquired	Field and lab sampling, chemical analysis
Data format	Raw, Analyzed
Description of data collection	Data were collected through the analysis of heavy metals in stormwater, which was collected in the field as influent and then applied to pervious concrete specimens after 28 days of curing. The pervious concrete mixtures were prepared with a water-to-cement (w/c) ratio of 32% and a sand ratio of 10%. Three different levels of palm oil kernel shell and seashell content, namely 0%, 25%, and 50%, were used as replacements for coarse aggregates. Additionally, two different single-size group were considered for both palm oil kernel shell and seashell: (6.3-9.5 mm) and (4.75-6.3 mm). The influent samples were collected from the surface runoff of stormwater. Heavy metal analyses were conducted on both influent and effluent using a PerkinElmer ELAN 6100 Series Inductively Coupled Plasma – Mass Spectrometer (ICP-MS).
Data source location	City/Town/Region: Taman University/Skudai/Johor: Country: Malaysia Latitude and longitude (and GPS coordinates, if possible) for collected samples/data: (1° 32′ 31.1604′' N 103° 37′ 47.64′' E)
Data accessibility	Repository name: Mendeley Data Data identification number: 10.17632/zw6wrx4xm7.2 Direct URL to data: https://data.mendeley.com/datasets/zw6wrx4xm7/2 No access control is required to view or download the
Related research article	E. Khankhaje, T. Kim, H. Jang, M. Rafieizonooz, Laboratory evaluation of heavy metal removal from stormwater runoff by pervious concrete pavement containing seashell and oil palm kernel shell, Constr. Build. Mater. 400 (2023) 132648. doi:10.1016/j.conbuildmat.2023.132648.

## 1 Value of the Data

• The presented experimental data show the extent of heavy metal removal from stormwater when pervious concrete pavement incorporates palm oil kernel shells and seashells as partial replacements for coarse aggregates.
• The data on the rate of heavy metal removal in pervious concrete incorporating seashells and palm oil kernel shells will help us comprehend the impact of these waste materials on heavy metal removal.
• The obtained data can be utilized for comparative analyses to assess the purification capabilities of pervious concrete pavements, thereby facilitating the production of more sustainable pervious concrete solutions.

## 2 Objective

The reason for collecting this dataset was the absence of a freely accessible dataset suitable for the development of sustainable pervious concrete and to investigate the impact of various waste materials on heavy metal removal in pervious concrete containing these waste materials. This dataset was created to record the removal of heavy metals (barium (Ba), selenium (Se), chromium (Cr), iron (Fe), cadmium (Cd), arsenic (As), and cobalt (Co)) from stormwater using pervious concrete pavements containing palm oil kernel shells and seashells. The measurement of heavy metals in stormwater is intended to assist engineers in comparing the effects of different waste materials on water purification, with the aim of finding the optimal mix design for pervious concrete containing waste materials to achieve more sustainable pervious concrete pavements with improved water purification capabilities. The provided heavy metal removal data were collected from stormwater samples taken in the field as influent and after passing through five pervious concrete specimens as effluent, for a total of ten events. These measurements were conducted using a PerkinElmer ELAN 6100 Series Inductively Coupled Plasma–Mass Spectrometer (ICP-MS). Furthermore, these datasets can supplement the results described in [1] by providing additional data on various heavy metals. This data can be utilized for the development, application, or comparison of other methods and the use of different waste materials for research purposes.

## 3 Data Description

**Fig. 1:**
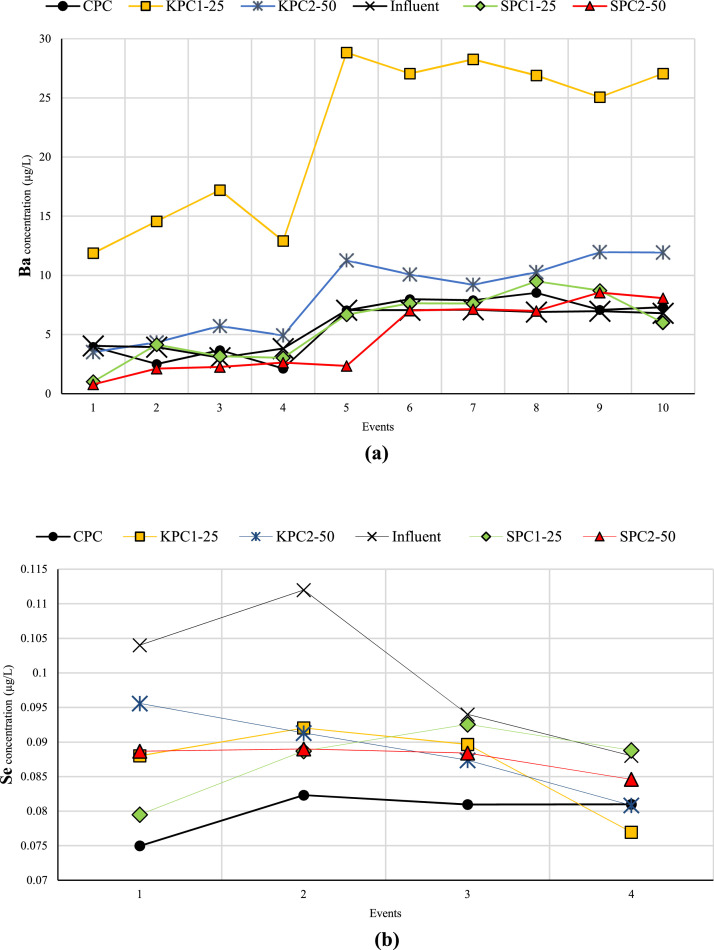

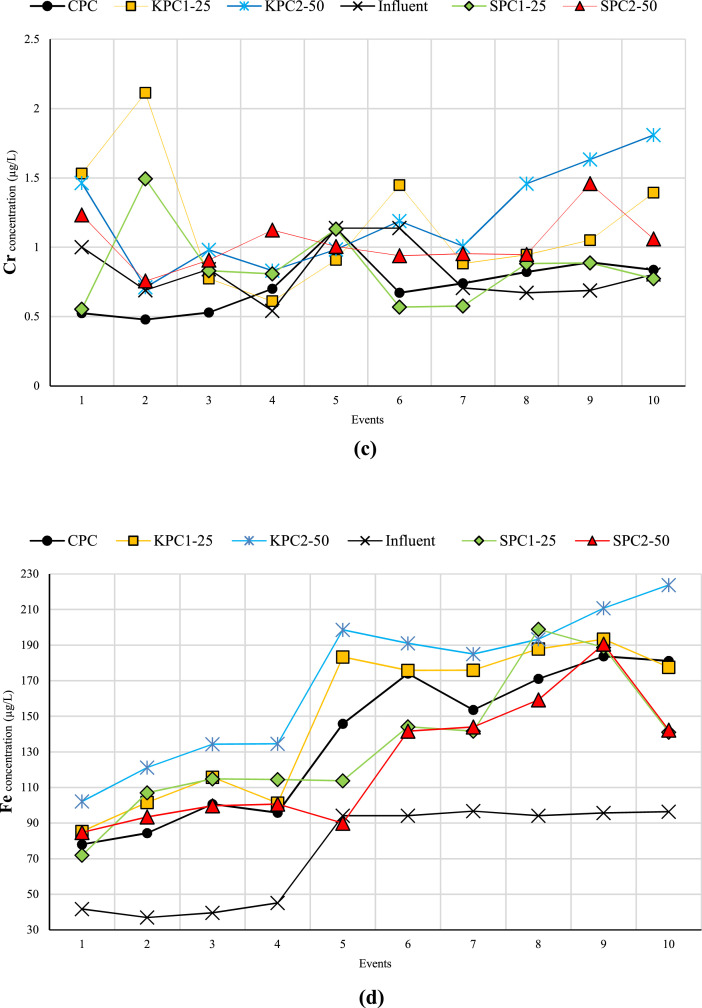

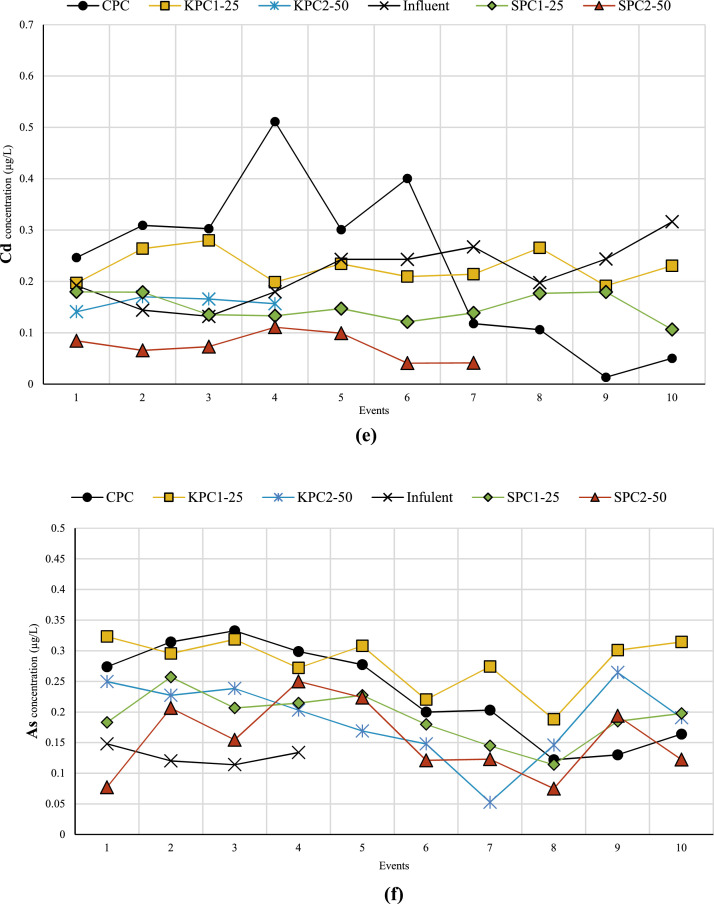

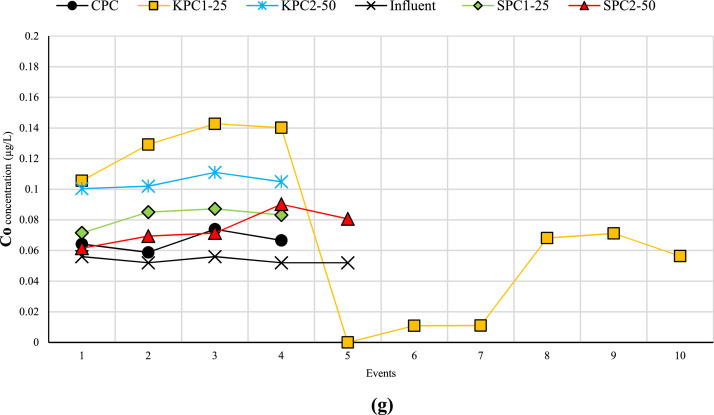
Concentration of heavy metals (a) Ba, (b) Se, (c) Cr, (d) Fe, (e) Cd, (f) As, and (g) Co in both effluent and influent for each specimen over ten events.

Fig. 1 displays the concentrations of heavy metals in both effluent and influent for each specimen over ten events. In several instances, the heavy metal concentration was below detection limits (ND), as shown in Fig. 1. Furthermore, Table 1 presents the heavy metals retained by each specimen during these ten events. Negative values indicate that the specimen was unable to remove the heavy metal, resulting in a higher concentration of heavy metal in the effluent compared to the influent. Additional data can be accessed in the supplementary file, available at the following URL: https://data.mendeley.com/datasets/zw6wrx4xm7/2. This file is provided in Excel format and comprises two separate sheets containing relevant information. The first sheet, titled "Raw Data," contains the raw data from the ICP-MS results, displaying the concentrations of heavy metals in both influent and effluent for each sample. The second sheet, titled "Analyzed Data," presents the amount of retained heavy metal in each sample based on the volume of influent and effluent. Additionally, the percentage of heavy metal removal by each sample is provided. For further data, please refer to [1,2].

**Table 1:** Retained heavy metal by each specimen for ten events.

Metal Retained (µg/L)							
	Cr	Co	Se	Ba	Fe	Cd	As
**CPC**	0.47521	−0.00826	0.02903	0.08907	−36.2674	−0.05433	−0.12587
	0.2112	−0.0068	0.02968	1.42208	−47.3682	−0.16512	−0.19416
	0.31224	−0.01792	0.01304	−0.62408	−61.0252	−0.17072	−0.21864
	−0.1582	−0.0146	0.007	1.688	−50.7528	−0.3312	−0.1648
	0.002266	ND	ND	0.017761	−51.6091	−0.05793	ND
	0.465876	ND	ND	−0.92721	−79.8285	−0.1576	ND
	−0.0327	ND	ND	−0.80965	−56.8555	0.149632	ND
	−0.15066	ND	ND	−1.61106	−76.8697	0.09189	ND
	−0.20208	ND	ND	−0.10896	−87.9799	0.230406	ND
	−0.03392	ND	ND	−0.50602	−84.6095	0.26598	ND

**KPC1-25**	−0.5312	−0.0496	0.016	−7.8356	−43.578	−0.0048	−0.1752
	−1.42338	−0.07721	0.01996	−10.6422	−64.6031	−0.11973	−0.17559
	0.06791	−0.08674	0.00433	−14.1582	−76.1607	−0.14799	−0.20442
	−0.06847	−0.08822	0.01105	−9.10705	−56.13	−0.01836	−0.13789
	0.230578	ND	ND	−21.7627	−89.2669	0.00893	ND
	−0.30952	ND	ND	−19.981	−81.6873	0.033582	ND
	−0.17547	ND	ND	−21.1567	−79.1985	0.05337	ND
	−0.27488	ND	ND	−19.9878	−93.6585	−0.06764	ND
	−0.36263	ND	ND	−18.0978	−97.6058	0.052483	ND
	−0.58961	ND	ND	−20.2515	−81.0697	0.085684	ND

**KPC2-50**	−0.93807	−0.04444	0.00842	0.5226	−60.4041	0.05106	−0.10148
	−0.23183	−0.05003	0.02071	−0.40338	−84.2306	−0.02605	−0.10733
	−0.45122	−0.05502	0.00664	−2.67826	−94.6907	−0.03362	−0.12442
	−0.13056	−0.05292	0.00716	−1.12096	−89.4742	0.02348	−0.06896
	0.152054	ND	ND	−4.20666	−104.454	ND	ND
	−0.51707	ND	ND	−3.023	−96.8892	ND	ND
	−0.26903	ND	ND	−2.11021	−88.3023	ND	ND
	−0.63612	ND	ND	−3.3647	−99.076	ND	ND
	−0.74265	ND	ND	−4.99264	−115.043	ND	ND
	−0.9711	ND	ND	−5.12302	−127.332	ND	ND

**SPC1-25**	0.44668	−0.01555	0.0245	3.02199	−30.1577	0.01233	−0.03485
	−0.80325	−0.03307	0.02331	−0.21833	−70.0928	−0.03519	−0.13702
	0.01074	−0.03122	0.00144	−0.12996	−75.1756	−0.00328	−0.09248
	−0.26645	−0.03125	−0.0008	0.7817	−69.3529	0.0468	−0.0806
	0.00753	ND	ND	0.38428	−19.5865	0.09578	ND
	0.569086	ND	ND	−0.56781	−50.0115	0.121606	ND
	0.130586	ND	ND	−0.52345	−44.9007	0.128644	ND
	−0.21049	ND	ND	−2.58874	−104.74	0.021127	ND
	−0.19752	ND	ND	−1.74512	−92.911	0.06452	ND
	0.029928	ND	ND	0.810664	−44.5865	0.20972	ND

**SPC2-50**	−0.23266	−0.00549	0.01534	3.25007	−42.9995	0.10763	0.07078
	−0.0665	−0.01742	0.023	1.81914	−56.4976	0.07814	−0.08648
	−0.0641	−0.0154	0.0056	0.781	−60.118	0.0589	−0.0407
	−0.58224	−0.03824	0.0034	1.18376	−55.599	0.06908	−0.11604
	0.13356	−0.02864	ND	4.71936	4.17256	0.14388	ND
	0.199923	ND	ND	0.041985	−47.4535	0.202113	ND
	−0.24718	ND	ND	−0.0485	−47.2582	0.22582	ND
	−0.27623	ND	ND	−0.07875	−65.0589	ND	ND
	−0.76968	ND	ND	−1.58026	−94.7736	ND	ND
	−0.25532	ND	ND	−1.27804	−45.7877	ND	ND

ND: Not detected.

## 4 Experimental Design, Materials and Methods

To create interconnected voids in pervious concrete, selecting single-sized aggregates is essential [3]. In this study, based on the sieve analysis results following ASTM C136 [4], Limestone (LS) was used as the natural coarse aggregate, with a single-size 6.30-9.5 mm, passing through the 9.5 mm sieve and retained on the 6.3 mm sieve. The oil palm kernel shell (KS) was collected from the Felda Kulai palm oil mill in Johor, Malaysia, and the cockle shells (CS) were obtained from waste generated by local restaurants in Pasir Putih, Pasir Gudang, on the south coast of Johor, Malaysia. Both the seashells and kernel shells were washed with tap water to remove traces of dirt. Ordinary Portland cement (OPC) was used in accordance with ASTM C150 [5] Type I, was used throughout the whole series of tests in the study. The water used in the preparation of all concrete mixtures was potable water. The specific gravity, water absorption, and resistance to degradation of coarse aggregates were tested in accordance with ASTM C127 and ASTM C131, respectively [6,7]. The specific gravity of the crushed limestone was measured to be 2.7 kg/m³, and it exhibited a water absorption rate of 1.8%. Naturally occurring river sand was sourced from the Sungai Sayong river quarry near Johor, Malaysia. This river sand was used as the fine aggregate in all the mixtures, with a maximum particle size of 4.75 mm, adhering to the demarcation specified in accordance with ASTM C33 [8]. The design of the investigated pervious concrete mixtures adhered to the guidelines presented in ACI 552R-10 [3], which was published in 2010. The pervious concrete mixes were formulated by incorporating cockle shells (CS) and palm kernel shells (KS) as replacements at three distinct levels: 0%, 25%, and 50%, serving as natural coarse aggregate replacements. Two different single-size categories of seashell and palm oil kernel shell were used: one with sizes ranging from 6.3 mm to 9.5 mm (passing through the 9.5 mm sieve and retained on the 6.30 mm sieve), and the other with sizes ranging from 4.75 mm to 6.3 mm (passing through the 6.3 mm sieve and retained on the 4.75 mm sieve). Throughout the experiment, the water-to-cement ratio (w/c) and the sand content remained constant at 32% and 10% (by weight percent of the coarse aggregate), respectively. In all mixtures, the amount of cement used was consistent at 339.5 kg per cubic meter. For a detailed description of the mixing process, please refer to [1]. Five cylindrical specimens (100 × 200 mm) were prepared for testing heavy metal removal, each following a specific mixture designation: CPC, SPC1-25, SPC2-50, KPC-25, and KPC2-50. Each mixture was assigned an individual code, where 'CPC,' 'SPC,' and 'KPC' represented control, cockle shell (CS), and palm kernel shell (KS) pervious concrete, respectively. The numbers ‘1’ and ‘2’ denoted the sizes of the waste materials used, specifically (6.3-9.5 mm) and (4.75-6.3 mm), respectively. Lastly, ‘25’ and ‘50’ indicated the percentages of waste material replacement in terms of the weight percentage of the natural coarse aggregate proportion. After a curing period of 28 days, the hardened pervious concrete specimens underwent heavy metal removal testing. The specimens were rinsed with tap water to remove any extraneous materials and then exposed to the laboratory atmosphere.

**Fig. 2:**
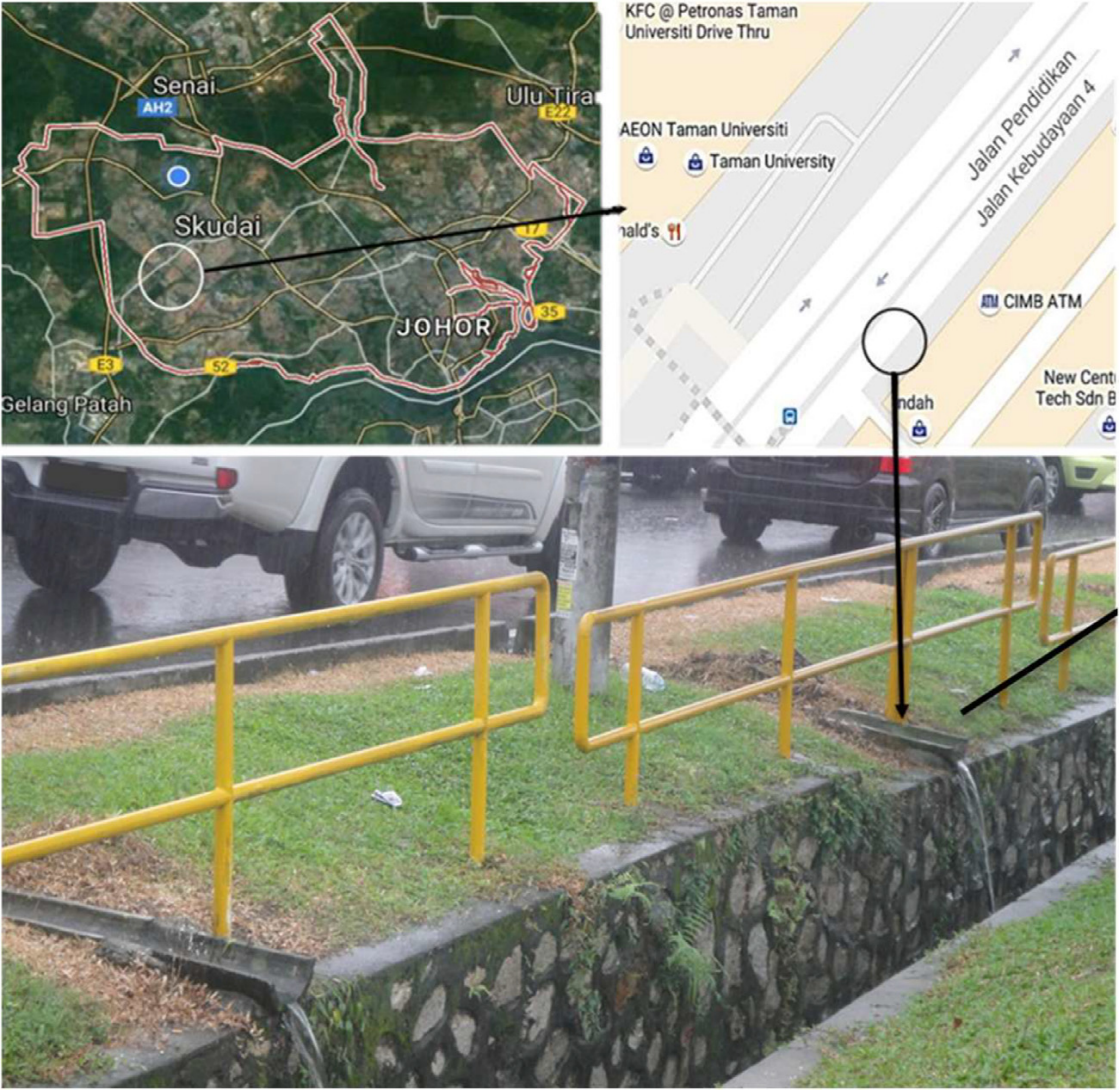
Sampling location of the stormwater.

**Fig. 3:**
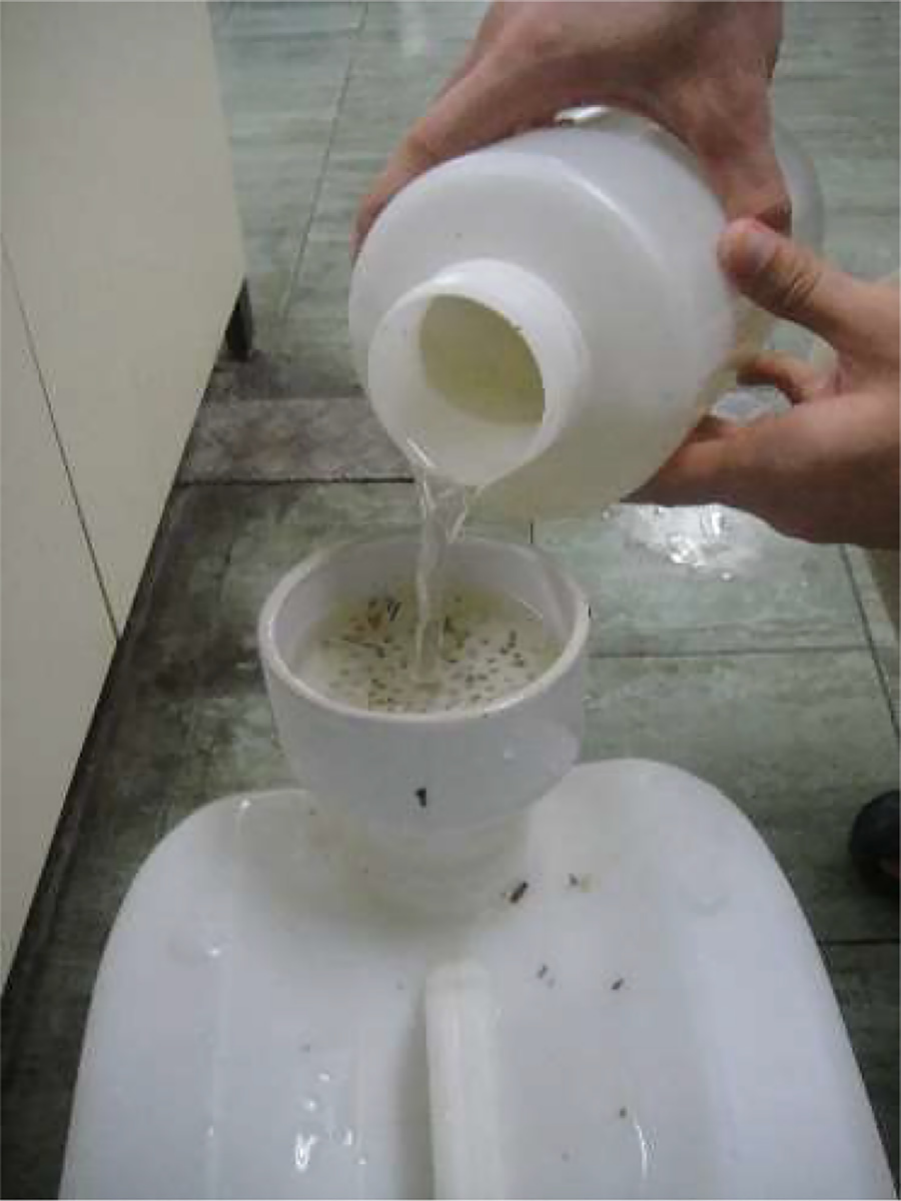
Filtering the sample.

Fig. 2 illustrates the sampling location for stormwater runoff, which served as the influent. The stormwater runoff was collected from a commercial area at Taman University, Skudai, Johor, located at coordinates 1° 32′ 31.1604′' N and 103° 37′ 47.64′' E, following the guidelines outlined in EPA 832-B-09-003 (2009) [9]. As depicted in Fig. 3, the stormwater sample underwent filtration, acidification, and was subsequently stored in high-density polyethylene (HDPE) bottles at a temperature of 4 °C for use as influent.

**Fig. 4:**
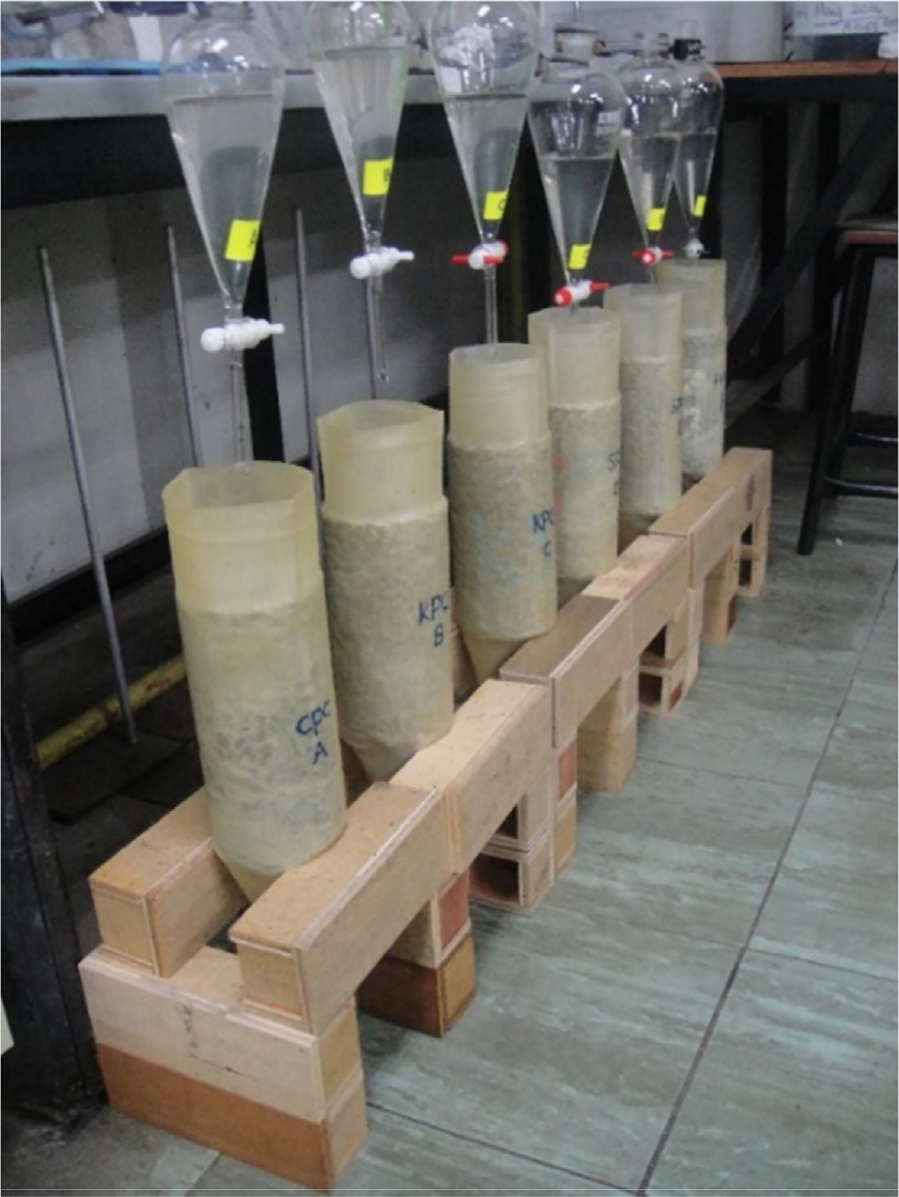
Arrangement for applying stormwater to the pervious concrete specimens.

Fig. 4 depicts the experimental setup for applying stormwater to the pervious concrete specimens. Before conducting the tests, all specimens were encased on their sides with a latex membrane to replicate a column setup, enabling water to be poured onto the tops and to exfiltrate through the bottoms of the specimens.

To facilitate the application of water to the concrete cylinders, a glass separating funnel was suspended above each concrete cylinder to allow the stormwater to drip through the cylinder. A glass beaker was positioned beneath the concrete cylinder to collect the effluent discharged from it. Effluent collection occurred approximately 5 minutes after the application of stormwater. Specific volumes (200 ml) of the prepared stormwater were applied to the cylinders, and the volume of effluent collected was recorded for mass balance purposes.

**Fig. 5:**
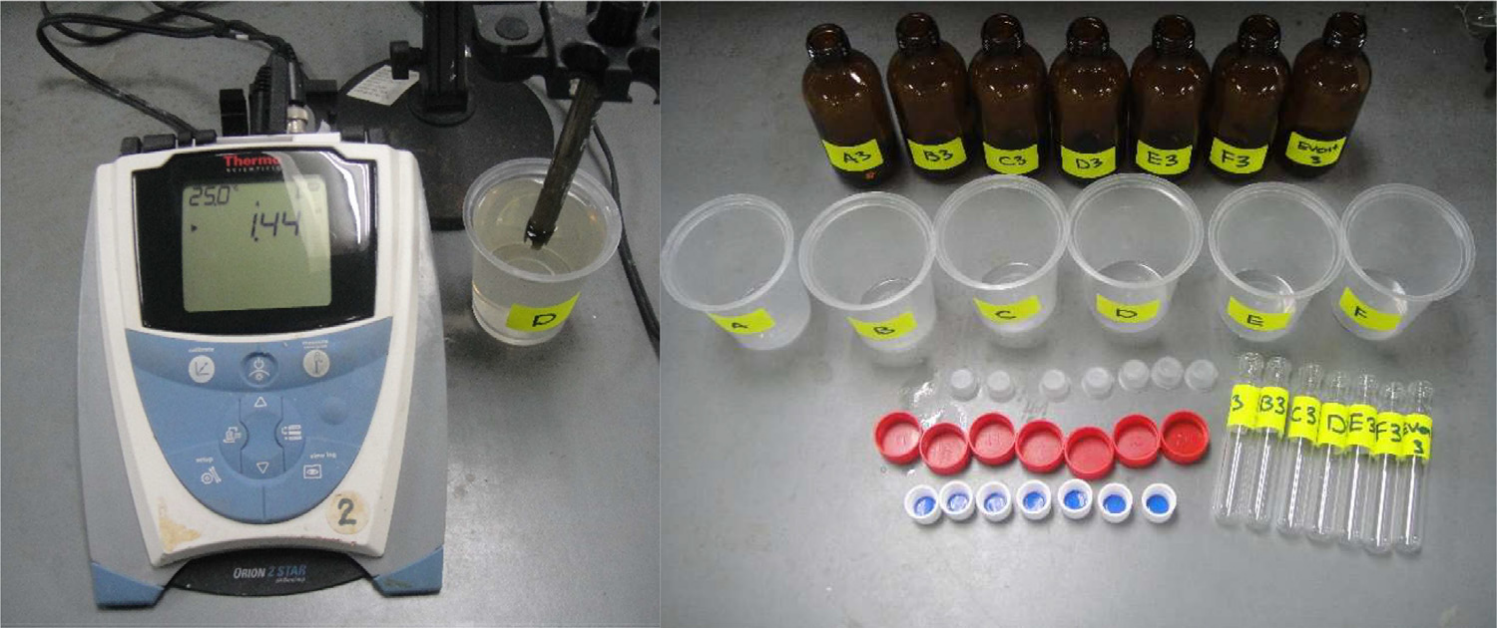
Acidifying (pH < 2) and storing effluent samples.

Individual effluent samples were collected from each cylinder for each event and were subsequently prepared separately for analysis. These samples were acidified to a pH below 2 using nitric acid and then stored in 30 mL glass culture tubes at 4 °C, as depicted in Fig. 5. Both influent and effluent samples were subjected to analysis for total dissolved zinc, copper, nickel, and lead using a PerkinElmer ELAN 6100 Series Inductively Coupled Plasma – Mass Spectrometer (ICP-MS). Following the analysis, the cylinders were dried in the laboratory for a minimum of 24 hours in preparation for the next event.

## Ethics Statements

Not applicable.

## CRediT authorship contribution statement

**Elnaz Khankhaje:** Methodology, Investigation, Writing – original draft. **Taehoon Kim:** Conceptualization, Methodology, Writing – review & editing. **Hyounseung Jang:** Conceptualization, Writing – review & editing, Supervision. **Chang-Soo Kim:** Writing – review & editing. **Jimin Kim:** Writing – review & editing. **Mahdi Rafieizonooz:** Writing – review & editing, Data curation.

## Declaration of Competing Interest

The authors declare that they have no known competing financial interests or personal relationships that could have appeared to influence the work reported in this paper.

## Data Availability

Dataset on the assessment of pervious concrete containing palm oil kernel shell and seashell in heavy metal removal from stormwater (Original data) (Mendeley Data)
